# Oxathiapiprolin, a Novel Chemical Inducer Activates the Plant Disease Resistance

**DOI:** 10.3390/ijms21041223

**Published:** 2020-02-12

**Authors:** Qin Peng, Zhiwen Wang, Pengfei Liu, Yinping Liang, Zhenzhen Zhao, Wenhui Li, Xili Liu, Ye Xia

**Affiliations:** 1 Department of Plant Pathology, College of Plant Protection, China Agricultural University, Beijing 100193, China; pengqin1991@126.com (Q.P.); frecawang@sina.com (Z.W.); pengfeiliu@cau.edu.cn (P.L.); 2 Department of Plant Pathology, College of Food, Agricultural, and Environmental Science, The Ohio State University, Columbus, OH 43210, USA; zhao.2047@buckeyemail.osu.edu; 3 College of Agronomy & Key Laboratory for Major Crop Diseases, Sichuan Agricultural University, Chengdu 611130, China; liangyinping3@163.com; 4 College of Horticulture and Landscape Architecture, Northeast Agricultural University, Harbin 150030, China; eau2014@163.com

**Keywords:** oxathiapiprolin, chemical inducer, plant disease resistance, callose, reactive oxygen species (ROS), salicylic acid (SA), jasmonic acid (JA)

## Abstract

Oxathiapiprolin was developed as a specific plant pathogenic oomycete inhibitor, previously shown to have highly curative and protective activities against the pepper Phytophthora blight disease under field and greenhouse tests. Therefore, it was hypothesized that oxathiapiprolin might potentially activate the plant disease resistance against pathogen infections. This study investigated the potential and related mechanism of oxathiapiprolin to activate the plant disease resistance using the bacterium *Pseudomonas syringae* pv *tomato* (*Pst*) and plant *Arabidopsis* interaction as the targeted system. Our results showed that oxathiapiprolin could activate the plant disease resistance against *Pst* DC3000, a non-target pathogen of oxathiapiprolin, in *Arabidopsis*, tobacco, and tomato plants. Our results also showed the enhanced callose deposition and H_2_O_2_ accumulation in the oxathiapiprolin-treated *Arabidopsis* under the induction of flg22 as the pathogen-associated molecular pattern (PAMP) treatment. Furthermore, increased levels of free salicylic acid (SA) and jasmonic acid (JA) were detected in the oxathiapiprolin-treated *Arabidopsis* plants compared to the mock-treated ones under the challenge of *Pst* DC3000. Besides, the gene expression results confirmed that at 24 h after the infiltration with *Pst* DC3000, the oxathiapiprolin-treated *Arabidopsis* plants had upregulated expression levels of the respiratory burst oxidase homolog D (*RBOHD*), JA-responsive gene (*PDF1.2*), and SA-responsive genes (*PR1*, *PR2*, and *PR5*) compared to the control. Taken together, oxathiapiprolin is identified as a novel chemical inducer which activates the plant disease resistance against *Pst* DC3000 by enhancing the callose deposition, H_2_O_2_ accumulation, and hormone SA and JA production.

## 1. Introduction

Plants encounter numerous biotic and abiotic factors during their growth and development. Among the biotic factors, plant pathogens are the ones that cause many destructive plant diseases which result in substantial yield and economic losses [1]. However, plants have evolved a complex innate defense system to prevent the infections of diverse pathogens [2,3]. Two levels of immune responses, including the pathogen/microbe-associated molecular patterns (PAMPs/MAMPs) triggered immunity (PTI) and effector-triggered immunity (ETI), were well studied in the past decades [2,3,4]. PTI is the first-level protection of plants, which is activated via the perception and recognition of PAMPs/MAMPs by the pattern recognition receptors (PRRs) of plants [5]. Different MAMPs can activate PTI, but the defense responses have similarities, including the cytoplasmic Ca^2+^ changes, mitogen-activated protein kinases (MAPKs) activation, reactive oxygen species (ROS) burst, increased level of nitric oxide (NO), enhanced callose deposition, upregulated defense-related genes, and phytoalexin production [6,7]. Although plants are protected by PTI, many pathogens can successfully infect plants via the inhibition of PTI through their effectors. Therefore, during the battles between plants and pathogens, plants have evolved another layer of plant immunity, effector triggered immunity (ETI), to protect themselves from pathogen infections. ETI usually leads to the hypersensitive response (HR). HR can be induced via the recognition and counteraction of microbial effectors, such as by the plant nucleotide-binding leucine-rich-repeat (NB-LRR) proteins. ETI also includes the plant systemic acquired resistance (SAR) at the non-pathogen infected sites to confer long-lasting disease resistance against diverse pathogens through priming effects [2,3].

Plants can activate the plant immunity under a variety of environmental stimuli, such as the attack of avirulent pathogens, colonization of beneficial microbes, and application of chemicals [8]. The plant disease resistance activated by avirulent pathogens, chemical inducers, and beneficial microbes can enhance plant defense against different pathogens, such as oomycetes, fungi, bacteria, and viruses [9,10,11,12]. The plant resistance activated by avirulent pathogens and chemical inducers is usually associated with strong defense responses, such as the enhanced accumulation of salicylic acid (SA) and the increased transcripts of pathogenesis-related (*PR*) genes [12,13,14]. For the wild-type *Arabidopsis* (Col-0) plants, the pre-inoculation with the avirulent *Pst* strain can activate the plant disease resistance via the accumulation of SA and transcripts of *PR1* [13]. Plant disease resistance can also be activated by the chemical inducers, such as SA, SA analog benzothiadiazole (BTH), and β-aminobutyric acid (BABA) [15]. Literature shows that the exogenous application of SA can induce plant disease resistance [16,17]. BTH can induce the plant disease resistance against many pathogenic infections in different plants, such as the *Erysiphe graminis* f. sp. *tritici* in *Triticum aestivum* [18], turnip crinkle virus, *Peronospora parasitica* and *Pseudomonas syringae* pathovar *tomato* (*Pst*) strain DC3000 (*Pst* DC3000) in *Arabidopsis* [19], *Fusarium oxysporum* f. sp. *radicis-lycopersic* in *Solanum lycopersicum* [20], and *Botrytis cinerea* in *Vitis vinifera* [21]. BABA, a nonprotein amino acid, can induce the plant disease resistance in many plants against different pathogens, such as the *B. cinerea* and *P. parasitica* in *Arabidopsis* [22,23], *Plasmopara viticola* in *Vitis vinifera* [24], and *Colletotrichum gloeosporioides* in *Mangifera indica* [25]. Plant disease resistance can also be activated by beneficial microbes. For example, the colonization of mycorrhizal fungus *Glomus mossae* in *S. lycopersicum* roots can activate the plant disease resistance against the pathogen *Phytophthora parasitica* [26,27].

*Pseudomonas syringae* (*Pst*) is a bacterial species widely used in studying the bacterial pathogenicity, plant–pathogen interactions, and pathogen ecology and epidemiology [28]. More than 50 pathovars have been identified in this species and each pathovar can infect a special group of host plants [28]. Collectively, the *Pst* pathovars can infect hundreds of different plants and result in diverse destructive diseases, including leaf spots and stem cankers [29]. The *Pst* strain DC3000 has the ability to infect both tomato and *Arabidopsis* plants, and its genome and genetic information have been well obtained [30]. Therefore, many researchers use this strain as a model to study the plant-bacteria interaction and the related disease resistance mechanism [31].

Oxathiapiprolin (OX), a piperidinyl thiazole isoxazoline fungicide, was synthesized by DuPont Company in 2007 [32,33]. OX was developed as an oomycete inhibitor with a high inhibition activity against many oomycetes, including *Phytophthora capsici, Phytophthora infestans, Phytophthora sojae, Phytophthora parasitica, Phytophthora nicotianae, Pseudoperonospora cubensis,* and *Pythium ultimum* [34,35,36,37]. Previous study showed that OX could interact with the oxysterol binding protein (OSBP) in oomycetes, which indicated that OSBP might be the target protein of OX [33]. Further study showed that in addition to the curative activity, OX also exhibited protective activity against the pepper Phytophthora blight under field and greenhouse tests [37]. Also, the protective activity of OX was better under the greenhouse conditions [37]. Because OX has better protective activity, we hypothesized that OX might have the potential to activate the plant disease resistance against the other pathogen infections in addition to oomycetes. To evaluate the potential function and investigate the related underlying mechanisms of OX in inducing plant disease resistance, the related experiments were conducted by mainly using the plant *Arabidopsis* and bacterium *Pst* DC3000 (a non-target bacterial pathogen of OX) interaction system. In addition, *Nicotiana benthamiana* (tobacco) and *Solanum lycopersicum* (tomato) were also utilized in the related study. For the outcome of the study, we identified that OX was a novel chemical inducer, which could activate the *Arabidopsis* plant disease resistance against *Pst* DC3000 through enhancing the callose deposition, H_2_O_2_ accumulation, and SA and JA accumulation.

## 2. Results

### 2.1. OX Induced the Plant Disease Resistance against Pst DC3000 Infection

**Figure 1:**
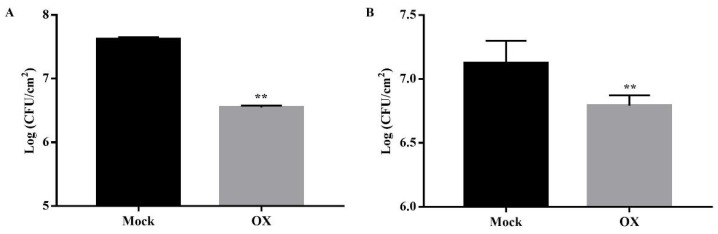
OX activated the plant disease resistance against *Pst* DC3000 infection in *Arabidopsis* plants. (**A**) OX activated the plant disease resistance against the *Pst* DC3000 infection via the spray treatment. The *Arabidopsis* plants (Col-0) were firstly sprayed with OX (40 μg/mL) or mock (water with DMSO). Then the *Arabidopsis* leaves were infiltrated with the *Pst* DC3000 (1 × 10^5^ Colony Forming Unit (CFU)/mL, OD_600_ = 0.0002) using a 1 mL syringe two days after the spray treatment. Three days after pathogen inoculation, the bacterial growth was quantified using the plate assay. (**B**) OX activated the plant disease resistance against the *Pst* DC3000 infection via pre-infiltration in local leaves. The lower leaves were pre-infiltrated with OX (40 μg/mL) or mock (water with DMSO) two days before the pathogen *Pst* DC3000 inoculation. The upper leaves were infiltrated with the *Pst* DC3000 (1 × 10^5^ CFU/mL, OD_600_ = 0.0002) using a 1 mL syringe. Three days after the pathogen inoculation, the bacterial growth was quantified using the plate assay. Significant differences between the OX-treated plants and the mock were indicated by the asterisks determined from the Student’s *t*-test (*p* < 0.05). Results shown (means ± SD) are from one of the three independent repeats with the consistent results.

To evaluate the potential role of OX in activating the plant disease resistance against the bacterial pathogen *Pst* DC3000, two types of treatments were designed and conducted. Firstly, the *Arabidopsis* plants were sprayed with OX (40 μg/mL) or mock (water with DMSO) to the leaves of the whole plants. Then the *Arabidopsis* leaves were challenged with the *Pst* DC3000 by the infiltration using a 1 mL syringe at two days after the pre-treatment of OX. The bacterial growth was examined at three days post infiltration (dpi). Compared to the mock plants, the *Pst* DC3000 growth was reduced significantly in the OX-treated plant leaves (Figure 1A). Furthermore, the lower three leaves of another set of *Arabidopsis* plants were firstly injected with OX (40 μg/mL) or mock (water with DMSO). Two days after the OX injection, the upper three or four *Arabidopsis* leaves were infiltrated with the *Pst* DC3000 with a 1 mL syringe. The bacterial growth was examined at 3 dpi. Compared to the mock plants, the *Pst* DC3000 growth was reduced significantly in the OX-treated plants (Figure 1B). 

Besides, OX-induced plant disease resistance against the bacterial pathogen *Pst* DC3000 was evaluated in tobacco and tomato plants. The tobacco and tomato plants were sprayed with OX (40 μg/mL) or mock (water with DMSO) on the leaves of the whole plants. Two days after the pre-treatment, the tomato or tobacco leaves were challenged with the *Pst* DC3000 by the infiltration with a 1 mL syringe. The bacterial growth was examined at 3 dpi. Compared to the mock plants, the *Pst* DC3000 growth was reduced significantly in the OX-treated tobacco or tomato leaves (Figure S1A,B), which was consistent with the results in *Arabidopsis*.

**Figure 2:**
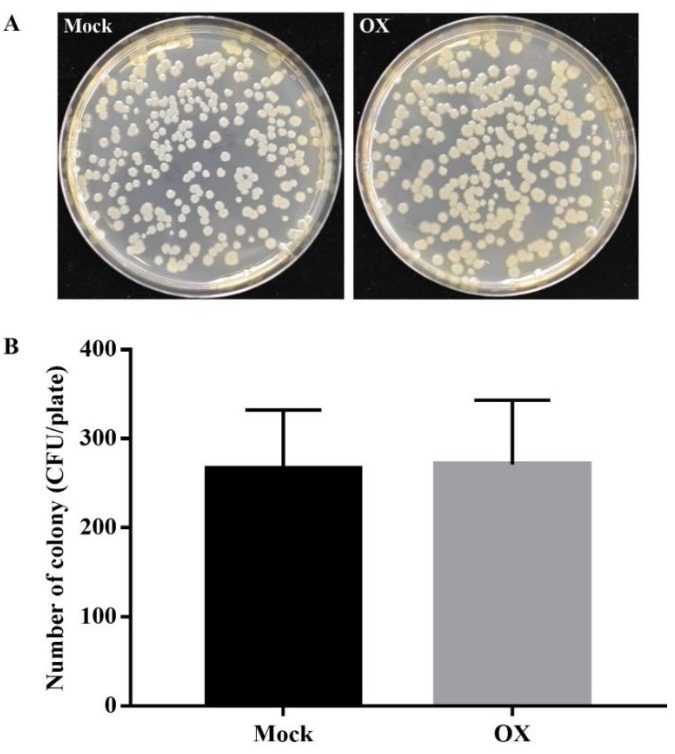
OX had no direct inhibition effect against the *Pst* DC3000 via the in-vitro plate assay. The direct inhibition effect of OX against the *Pst* DC3000 was performed by using King’s B agar amended with mock (DMSO) or OX (40 µg/mL). About 100 µL bacterial suspension (5 × 10^3^ CFU/mL) was uniformly plated on the surface of the agar plate. The photos of the plates (**A**) were taken, and the colony number (**B**) was counted after 2 days of darkness-incubation at 28 °C. Three replicates were prepared for each treatment. Results shown (means ± SD) are from one of the three independent repeats with the consistent results.

To further confirm that the reduced growth of bacteria was due to the OX-induced plant disease resistance, the direct inhibition effect of OX against the *Pst* DC3000 was tested by the in-vitro plate assay. The results showed that OX had no direct inhibition effect against the *Pst* DC3000 (Figure 2A,B). All these results indicated that OX could induce the plant disease resistance against the *Pst* DC3000 in *Arabidopsis*, tomato, and tobacco plants.

### 2.2. OX Activated the Plant Disease Resistance by Enhancing the H_2_O_2_ Accumulation in Arabidopsis Plants

**Figure 3:**
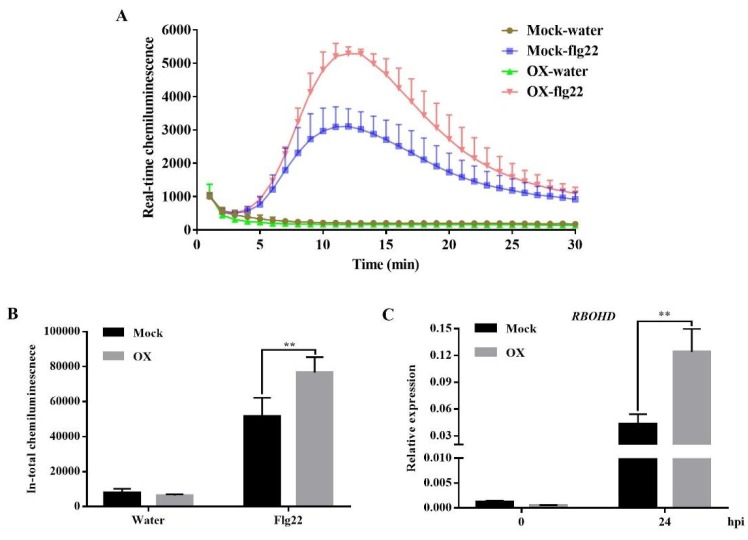
The OX enhanced H_2_O_2_ accumulation in *Arabidopsis* plants upon the flg22 or *Pst* DC3000 treatments. Wild-type *Arabidopsis* plants (Col-0) were sprayed with OX (40 μg/mL) or mock (water with DMSO). Two days later, the H_2_O_2_ accumulation in leaves was continuously detected for 30 min using the GLOMAX 20/20 luminometer (Promega). (**A**) The real-time chemiluminescence in the leaves and (**B**) total chemiluminescence in the leaves within 30 min of flg22 treatment. (**C**) The relative expression levels of *RBOHD* in mock- and OX-treated *Arabidopsis* leaves before and after the *Pst* DC3000 inoculation. *ACTIN* was used as the internal reference gene. Significant differences between the OX-treated plants and the mock were indicated by the asterisks determined from the one-way analysis of variance (ANOVA) statistical test using SPSS ver. 21 (*p* < 0.05). Results shown (means ± SD) are from one of the two independent repeats with the consistent results.

To investigate the underlying mechanism of OX-induced plant disease resistance, the H_2_O_2_ accumulation was compared between the mock and OX-treated *Arabidopsis* plants. For both real-time and total quantification of H_2_O_2_, there were no significant differences in the chemiluminescence accumulation between the mock- and OX-treated *Arabidopsis* without the flg22 treatment (a peptide corresponding to the most conserved domain of the bacterial flagellin, acting as a common and potent elicitor of PTI). However, the chemiluminescence accumulation was significantly increased in the OX-treated *Arabidopsis* plants with the flg22 treatment compared to the mock-treated ones (Figure 3A,B). The result indicated the enhanced ROS burst in the OX-treated *Arabidopsis* plants. Further experiments were conducted to quantify the gene expression level of the ROS related gene, such as the respiratory burst oxidase homolog D (*RBOHD*), which plays a crucial role in the accumulation of ROS in apoplast [38]. The qPCR result showed that the transcription level of *RBOHD* was not significantly changed between the mock- and OX-treated *Arabidopsis* plants before the *Pst* DC3000 treatment. However, the OX-treated *Arabidopsis* plants had significantly higher expression level of *RBOHD* than that of the mock-treated ones at 24-h post infiltration (hpi) with the *Pst* DC3000 (Figure 3C), which was consistent with the increased H_2_O_2_ accumulation in response to the flg22 treatment. All these results indicated that OX induced the plant disease resistance potentially by enhancing the H_2_O_2_ accumulation in *Arabidopsis* plants upon the pathogen *Pst* DC3000 inoculation.

### 2.3. OX Activated the Plant Disease Resistance by Increasing the Callose Deposition in Arabidopsis Plants

**Figure 4:**
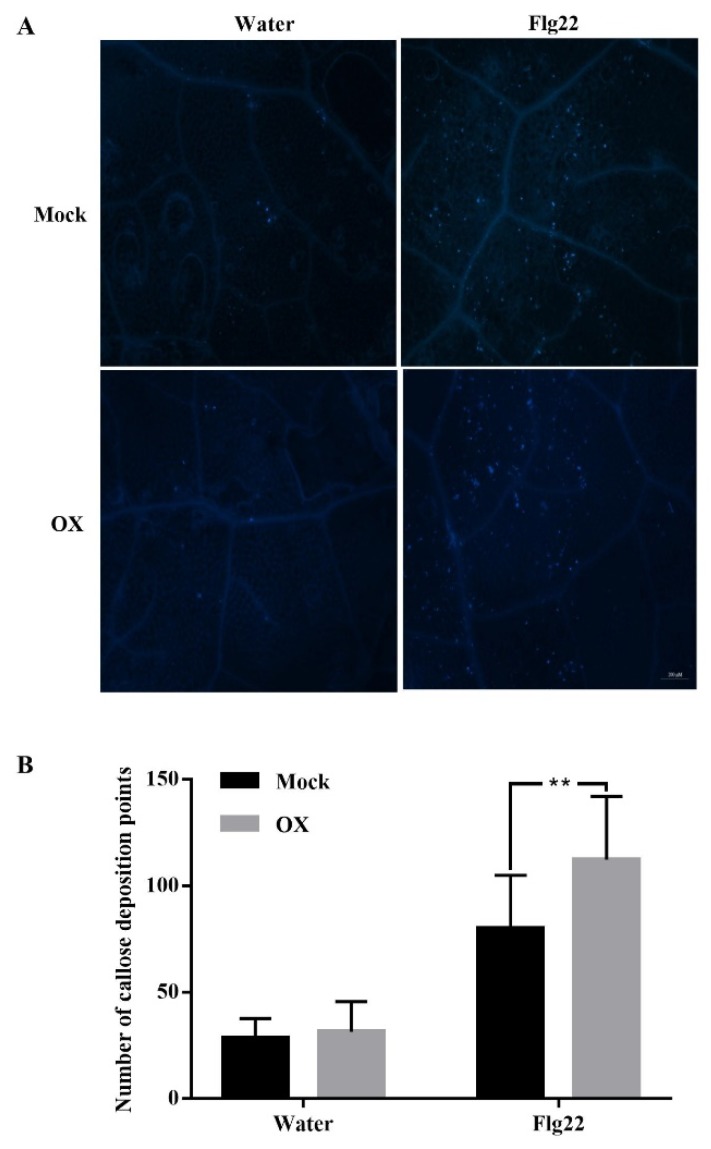
OX enhanced the callose deposition in *Arabidopsis* plants. Wild-type *Arabidopsis* plants (Col-0) were firstly sprayed with OX (40 μg/mL) or mock (water with DMSO). Then the *Arabidopsis* leaves were treated with water or 100 μM flg22 using the infiltration with a 1 mL syringe two days after the spray treatment. The leaves were then stained using 0.01% aniline blue for the callose deposition after 14–16 hpi of flg22 treatment. (**A**) The images of callose deposition in *Arabidopsis* leaves were taken by a Nikon Eclipse 80i epi-fluorescent microscope (Nikon, Tokyo, Japan). (**B**) The number of callose deposition points was counted by the Image J software. Significant differences between the OX-treated plants and the mock-treated plants were indicated by the asterisks determined from the Student’s *t*-test (*p* < 0.05). Results shown (means ± SD) are from one of the two independent repeats with consistent results.

To further investigate the underlying mechanism of OX-induced plant disease resistance, the callose deposition, which is an indicator of cell wall defense [39,40], was compared between the mock- and OX-treated *Arabidopsis* plants (Figure 4A,B). The callose deposition levels were low and similar between the mock- and OX- treated *Arabidopsis* plants with the water treatment. While the callose deposition was significantly increased in both the mock- and OX-treated *Arabidopsis* plants with the flg22 treatment. However, the callose deposition was significantly higher in the OX-treated *Arabidopsis* plants than the mock-treated ones after the flg22 treatment, indicating an enhanced cell wall-related defense response in the OX-treated *Arabidopsis* plants during the interactions with the bacterial pathogen.

### 2.4. OX Activated the Plant Disease Resistance by Enhancing both the SA and JA Related Pathways in Arabidopsis Plants

**Figure 5:**
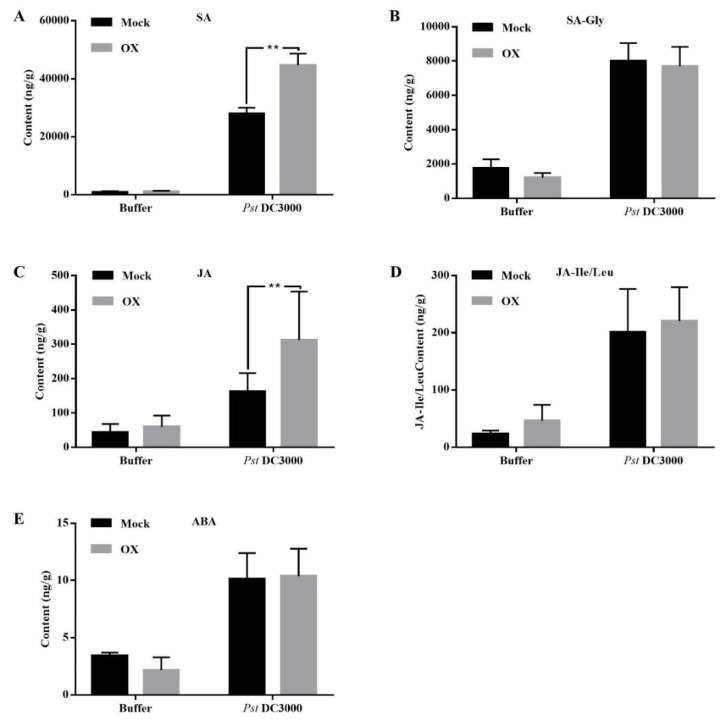
OX increased the levels of free SA and JA in *Arabidopsis* plants with the *Pst* DC3000 treatment. (**A**) Free SA level. (**B**) SA-Gly (glycosylated derivatives of salicylic acid) level. (**C**) Free JA level. (**D**) JA-Ile/Leu (JA-isoleucine/leucine) level. (**E**) ABA level. Wild-type *Arabidopsis* plants (Col-0) were sprayed with OX (40 μg/mL) or mock (water with DMSO). The *Arabidopsis* leaves were infiltrated with the *Pst* DC3000 (5 × 10^5^ CFU/mL, OD_600_ = 0.001) with a 1 mL syringe two days after the spray treatment. The samples were collected for hormone quantification at 2 dpi. Significant differences between the OX-treated plants and the mock were indicated using the asterisks determined from the Student’s *t*-test (*p* < 0.05).

To further investigate the underlying mechanism of OX-induced plant disease resistance, the levels of several plant hormones in the mock- and OX-treated *Arabidopsis* plants before and after the *Pst* DC3000 inoculation were quantified (Figure 5). The levels of free SA and JA did not show significant differences in the mock- and OX-treated *Arabidopsis* plants without the challenge of *Pst* DC3000. However, compared to the mock-treated ones, both free SA and free JA levels were significantly higher in the OX-treated *Arabidopsis* plants under the challenge of the *Pst* DC3000 (Figure 5A,C). The levels of SA-Gly (glycosylated derivatives of salicylic acid), JA-Ile/Leu, and ABA had no significant differences in the mock- and OX-treated *Arabidopsis* plants with or without the *Pst* DC3000 inoculation (Figure 5B,D,E). The hormone results indicated that OX activated the plant disease resistance by enhancing the accumulation of both SA and JA.

**Figure 6:**
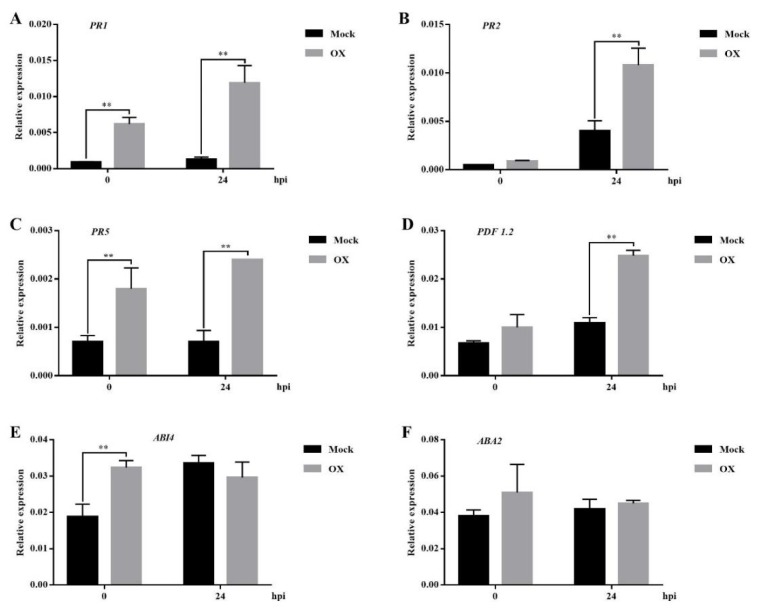
OX enhanced the transcription levels of the SA- and JA-responsive genes in *Arabidopsis* plants with the *Pst* DC3000 treatment. Wild-type *Arabidopsis* plants (Col-0) were sprayed with OX (40 μg/mL) or mock (water with DMSO). The *Arabidopsis* leaves were infiltrated with the *Pst* DC3000 (1 × 10^5^ CFU/mL, OD_600_ = 0.0002) with a 1ml syringe two days after the spray treatment. Samples for RNA extraction were collected and frozen in liquid nitrogen at 0 and 24 hpi. The relative transcription levels of *PR1* (**A**), *PR2* (**B**), *PR5* (**C**), *PDF1.2* (**D**), *ABI4* (**E**) and *ABA2* (**F**) were quantified by real-time qPCR. *ACTIN* was used as the internal reference gene. Significant differences between the OX-treated plants and the mock-treated plants were indicated by the one-way ANOVA statistical test by SPSS ver. 21 (*p* < 0.05). Results shown (means ± SD) are from one of the two independent repeats with the consistent results.

Further, the transcription levels of several typical genes in SA and JA related pathways were quantified. First, the transcription levels of SA-responsive genes were analyzed, including *PR1*, *PR2*, and *PR5*. Although SA accumulation did not differ significantly without the *Pst* DC3000 inoculation, the significantly higher transcription levels of *PR* genes were shown in the OX-treated *Arabidopsis* plants compared to the mock-treated ones, except *PR2* (Figure 6A–C). Consistent with the hormone results, the OX-treated *Arabidopsis* plants had significantly higher expression levels of these three *PR* genes than the mock-treated ones at 24 hpi with the *Pst* DC3000 treatment (Figure 6A–C). Next, a typical JA-responsive gene *PDF1.2* was analyzed, which is important for the defense against the necrotrophic pathogens [41]. The result showed that the expression level of *PDF 1.2* was significantly higher in the OX-treated *Arabidopsis* plants than the mock-treated ones at 24 hpi with the *Pst* DC3000 treatment (Figure 6D), which was consistent with the JA accumulation. Consistent with the similar accumulation of ABA with or without the challenge of *Pst* DC3000, the transcription levels of two ABA related genes, *ABI4* and *ABA2*, showed the similar levels in both the OX-treated and mock-treated *Arabidopsis* plants at 24 hpi with the *Pst* DC3000 inoculation (Figure 6E,F). Taken together, both SA- and JA-signaling pathways might contribute to the OX-induced plant disease resistance in *Arabidopsis*, while ABA might not be very important for the OX-induced plant disease resistance.

## 3. Discussion

OX was firstly synthesized as a fungicide to control oomycete-associated plant diseases by the DuPont Company in 2007 [32,33]. Previous research indicated that OX exhibited direct and high inhibition activity against the growth of many oomycete pathogens with a low dose. The target protein of OX was the oxysterol binding protein (OSBP) in oomycetes [33,34,35,36,37]. Besides, OX exhibited both curative and protective activity against pepper Phytophthora blight, and the protective activity was better [37]. There is no further study on the potential of OX to induce the plant immunity against the other pathogens in addition to oomycete pathogens. Therefore, in the present study, the related responses and mechanisms of the induced plant disease resistance by OX was investigated mainly using the *Pst* DC3000 and *Arabidopsis* interaction system.

The *Pst* DC3000 and *Arabidopsis* interaction system is widely used in studying the interactions of pathogens and hosts [42]. Here, we found that spraying the *Arabidopsis* plants with OX at the concentration of 40 μg/mL two days before the pathogen *Pst* DC3000 inoculation could enhance the *Arabidopsis* plant defense against the *Pst* DC3000. A further experiment showed that the infiltrated local leaves of *Arabidopsis* with OX could enhance the defense of the systemic leaves against *Pst* DC3000. Besides, spraying treatment of the plants with OX at 40 μg/mL could activate the plant disease resistance against the *Pst* DC3000 in tobacco and tomato plants. All these results indicated that OX could serve as a common inducer for plant immunity to activate the plant disease resistance against the *Pst* DC3000 infection in diverse plant species.

Reactive oxygen species (ROS) are widely distributed in plants under different conditions, including the challenges of both abiotic and biotic stresses. ROS are important factors to regulate the cellular process and signaling transduction in plants. ROS also play important roles in plant immune response against the pathogen infections [43]. Under the attack of pathogens, plants will activate defense responses, such as the oxidative burst to produce ROS at the infection site, which is one of the earliest events of a plant’s defense responses [44]. Various subcellular compartments of plants can produce ROS, including the mitochondria, chloroplasts, cell wall, plasma membrane, and peroxisomes/glyoxysome [43]. However, the apoplastic ROS, mainly produced at the plasma membrane, are considered as the major ones during the interactions of plants and pathogens [43]. The NADPH oxidases, a member of the respiratory burst oxidase homolog (RBOH) family, are very important in ROS production. They can transfer electrons from cytosolic NADPH or NADH to the apoplastic oxygen and finally involve in the production of hydrogen peroxide (H_2_O_2_) [43,44,45]. In *Arabidopsis*, there are 10 members of RBOHs, among which RBOHD is the most studied and plays an important role in plant immunity [38,45]. In this study, the levels of H_2_O_2_ were quantified using the Glomax 20/20 single-well luminometer (Promega), and the transcription level of *RBOHD* was quantified using real-time qPCR. Compared to the mock-treated *Arabidopsis* plants, the H_2_O_2_ accumulation and the transcription level of *RBOHD* were significantly increased in the OX-treated *Arabidopsis* plants with the flg22 treatment or the *Pst* DC3000 inoculation. The results indicated that the ROS burst may contribute to the OX-induced plant disease resistance.

The plant cell wall is one of the first barriers of plants to prevent themselves from the pathogen infections [46]. Plants can enhance the depositions of several polymers, such as employ the toxic compounds, phenolic complexes, and callose to defend themselves against pathogen infections [47]. Among these polymers, callose is a polymer with a high–molecular weight, and it consists of β-(1,3)-glucan, which can be induced by the PAMPs, such as flg22 [39]. The callose deposition is a judging method widely used to evaluate the plant PTI [39,40]. In this study, the callose deposition was visualized using the aniline blue staining based on the previous report [40]. Compared to the mock-treated *Arabidopsis* plants, the callose deposition was significantly higher in the OX-treated *Arabidopsis* plants under the induction of flg22, indicating a strengthened cell wall in the OX-treated *Arabidopsis* plants, which might contribute to the OX-induced plant disease resistance.

Plant hormones are important for the development and defense of plants. There are nine major plant hormones. Among them, salicylic acid (SA), jasmonic acid (JA), and ethylene (ET) are the main hormones to regulate plant immunity under different biotic and abiotic stresses [48,49]. These three main pathways could cross-talk with the other hormone pathways to confer resistance against diverse pathogen infections [48,49]. Generally, SA is associated with the plant defense against the hemi-biotrophic and biotrophic pathogens. In contrast, JA plays a more important role in plant defense against the necrotrophic pathogens [48]. The functions of SA and JA in plant immunity could be antagonistic [49,50]. However, literature has shown that SA and JA could cooperate with each other and function synergistically in plant immunity [51,52,53]. In general, to defend against the infections of the necrotrophic and biotrophic pathogens, ABA is a negative regulator in plants [49]. However, there is evidence showing that ABA could also be a positive regulator of plant defense, such as *Arabidopsis* plant resistance against the *Pst* DC3000 (a hemi-biotrophic pathogen) at the early stage, by inducing plant stomata closure [54]. Therefore, the exact function of ABA in plant defense should be determined by different plant-pathogen interactions at different infection stages [49]. 

Plant hormones are very important in plant defense. Whether the hormone pathways contributed to the OX-induced plant disease resistance remains unclear. In this study, we found that under the challenge of the *Pst* DC3000, both the free SA and JA levels were significantly increased in the OX-treated *Arabidopsis* plants than those of the mock-treated *Arabidopsis* plants at 48 hpi. The levels of SA-Gly, JA-Ile/Leu, and ABA had no significant differences in the mock- and OX-treated *Arabidopsis* plants. In addition, the real-time qPCR results showed that the OX-treated *Arabidopsis* plants had higher transcription levels of three *PR* genes, including *PR1, PR2,* and *PR.* The level of the JA-responsive gene, *PDF 1.2,* was also significantly enhanced compared to the mock-treated *Arabidopsis* plants. However, the plants showed similar transcription levels of two ABA signaling genes, *ABI4* and *ABA2*. Taken together, we discovered that both SA and JA pathways were activated in the OX-treated *Arabidopsis* plants upon the *Pst* DC3000 inoculation, which indicated that both SA- and JA-signaling pathways might contribute to the OX-induced plant disease resistance in *Arabidopsis* and these pathways might work synergistically. However, the ABA pathway might not be significantly involved in the related plant disease resistance.

The mechanisms of the OX-induced and the BTH or BABA-induced plant disease resistance have similarities and differences. Literature shows that BTH or BABA can induce the plant broad-spectrum disease resistance [19,20,21,22,23,24,25]. For instance, BTH can induce the plant disease resistance against turnip crinkle virus, *Peronospora parasitica,* and *Pst* DC3000 in *Arabidopsis* [19]. BABA can induce the plant disease resistance against *B. cinerea* and *P. parasitica* in *Arabidopsis* [22,23]. For the present study, we found that OX could induce the plant disease resistance against the *Pst* DC3000 in *Arabidopsis*, tobacco and tomato plants. Whether OX could induce the plant broad-spectrum disease resistance against other pathogens needs to be further investigated. Besides, the mechanisms of BTH or BABA-induced plant disease resistance are complicated and dependent on the systems of the plant-pathogen interaction. For example, BTH can induce the plant disease resistance against *B. cinerea* by modifying the amino acid profile in grape [55]. BABA can induce the plant disease resistance against *Plasmopara viticola* by enhancing the callose formation and JA signaling or by the accumulation of lignin in grape plants [24,56]. Both BTH and BABA can induce the plant disease resistance against the *Pst* DC3000, mainly by activating the SA signaling pathway, such as the increased accumulation of SA and PR proteins [19,23]. However, in the present study, we found that OX activated the plant disease resistance against the *Pst* DC3000 by increasing the accumulation of both SA and JA and their responsive genes. Whether the OX-induced plant disease resistance is dependent on both SA and JA signaling pathways needs to be further studied.

In summary, in this study, we discovered that OX was a novel chemical inducer to activate the plant disease resistance against the *Pst* DC3000 in *Arabidopsis* by enhancing the callose deposition, H_2_O_2_ accumulation, and SA and JA production and related signaling pathways. Future research can be carried out to investigate the potential and associated mechanisms of OX as an important plant disease resistance inducer against other plant pathogens, such as bacteria, fungi, oomycetes, and viruses in different plant species. Such research will facilitate our understanding of the mechanism of the induced plant disease resistance and its application in agriculture to enhance plant health against diverse pathogen infections. 

## 4. Materials and Methods

### 4.1. Plant Growth Conditions

Wild-type *Arabidopsis thaliana* (Col-0) plants and tobacco (*Nicotiana benthamiana*) plants were cultured in a growth room at 22 °C with 10 h light/14 h darkness for *Arabidopsis thaliana* (Col-0) and 16 h light/8 h darkness for tobacco, respectively. Tomato (*Solanum lycopersicum*) plants (cv. OH88119) were cultivated in a greenhouse at room temperature with 12 h light/12 h darkness. The pathogen inoculation experiments were conducted 4–5 weeks after the transplanting.

### 4.2. The OX Treatment, Pst DC3000 Infiltration, and Bacteria Growth Assay

OX, from DuPont Crop Protection (Wilmington, DE, USA), was dissolved in DMSO for a stocking solution at 4 × 10^4^ μg/mL. The stocking solution was 1000 times diluted in water for a final concentration of 40 μg/mL. To evaluate the potential role of OX in activating the plant disease resistance against the pathogen *Pst* DC3000 infection, the *Arabidopsis,* tobacco, and tomato plants were sprayed with OX solution (40 μg/mL) or mock (water containing the same amount of DMSO) on leaves firstly. Two days after the spray treatment, the leaves were infiltrated with 10 mM MgCl_2_ containing the *Pst* DC3000 using a 1 mL syringe. The bacteria concentration was 1 × 10^5^ CFU/mL (OD_600_ = 0.0002). Another test for the OX-induced plant disease resistance in *Arabidopsis* was through the treatment of the local leaves. For example, the lower leaves of *Arabidopsis* plants were infiltrated with the OX solution (40 μg/mL) or mock firstly. Two days later, the upper three or four leaves were infiltrated with bacterial broth, as described above. Three days after the infiltration with the *Pst* DC3000, three leaf discs were collected from the bacteria infiltrated leaves as one sample for the bacteria growth assay [57]. Each treatment had at least three replicates, and the whole experiment was conducted three times with consistent results.

### 4.3. The Direct Inhibition Effect of OX against the Pst DC3000 by the in-Vitro Plate Assay

The direct inhibition effect of OX against the *Pst* DC3000 was performed by the in-vitro plate inhibition assay. King’s B agar amended with 40 µg/mL OX and the concentration of DMSO was limited to 0.1% (*v*/*v*). An equivalent concentration of DMSO was used for the negative controls. Bacterial suspension (100 µL, 5 × 10^3^ CFU/mL) was uniformly plated on the agar surface. The colony number was counted after 2 days of darkness-incubation at 28 °C. Three replicates were prepared for each treatment, and the entire experiment was performed three times with consistent results.

### 4.4. The H_2_O_2_ Detection and Callose Deposition

The H_2_O_2_ was detected according to a previous report with a minor modification [58]. The mock or OX-treated *Arabidopsis* leaves were sliced into 5 mm diameter discs and incubated in the plastic plates containing the distilled water for 12 h. Three leaf discs were treated with water or 200 nM flg22 (GeneScript, Piscataway, NJ, USA) as the PAMP treatment in 200 μL buffer containing 20 μM luminol and 1 μg/mL horseradish peroxidase. Luminescence was continuously measured for 30 min by using the Glomax 20/20 single well luminometer (Promega, Madison ,WI, USA). Each treatment had at least three replicates and the whole experiment was conducted twice with the consistent results.

The callose deposition was measured based on a protocol reported by Lin Jin [40]. In brief, the mock or OX-treated *Arabidopsis* plants were infiltrated with 100 μM flg22 or water. The infiltrated leaves were stained for callose by 0.01% aniline blue (Cat #: 415049, Sigma-Aldrich, St. Louis, MO, USA) after 14–16 h after infiltration (hpi). The callose deposition was visualized by a Nikon Eclipse 80i epi-fluorescent microscope (Nikon, Tokyo, Japan) and further quantified by Image J software (http://rsbweb.nih.gov/ij/). Each treatment included six plants, and the whole experiment was conducted twice with consistent results.

### 4.5. Plant Hormone Extraction and Quantification

Plant hormones were extracted and detected according to the previous reports [59,60]. In brief, the mock or OX-treated *Arabidopsis* plants were infiltrated with buffer (10 mM MgCl_2_) or buffer containing the *Pst* DC3000 cells. The bacterial cell concentration was 5 × 10^5^ Colony Forming Unit (CFU)/mL (OD_600_ = 0.001). Two days after infiltration, the leaf samples (approximately 110 mg fresh weight per sample) were collected and extracted with 400 μL extraction buffer containing 10% methanol and 1% acetic acid. Isotope labelled internal standards (d_4_-SA, d_5_-JA, and d_6_-ABA) were added at the beginning of the extraction. The amounts of the internal standards added were 1 ng of ^2^H6-ABA (d_6_-ABA, Toronto Research Chemicals, North York, ON, USA; part #: A110002), 15 ng ^2^H4-SA (d_4_-SA, CDN Isotopes, Pointe-Claire, QC, Canada, part #: D-1156), and 150 ng ^2^H5-JA (d_5_-JA, CDN Isotopes, Pointe-Claire, QC, Canada, part #D-6936). After the addition of the extraction buffer, the tubes were incubated on ice for 30 min and centrifuged at 4 °C (13,000 g, 10 min). The supernatant was collected and re-extracted. After two procedures of extraction with the extraction buffer, the final supernatant was collected and analyzed using UPLC/ESI/MS with the Thermo Fisher Ultimate 3000 system (Thermo Fisher, Waltham, MA, USA). The UPLC separation was carried out on a 3 μm C18 (100 mm × 2.0 mm) column (Waters company, Milford, MA, USA) at 35 °C. The mobile phase was set for a continuum gradient from (94.9% H_2_O: 5% CH_3_CN: 0.1% CHOOH) to (5% H_2_O: 94.9% CH_3_CN: 0.1% CHOOH) over 20 mins. The analysis of the compounds was based on Multiple Reaction Monitoring (MRM) of ion pairs for the labelled and endogenous hormones. Transition settings for SA, JA, and ABA were ^2^H_4_-SA 141 (97), SA 137 (93), ^2^H_6_-ABA 269 (159), ABA 263 (153), SA-Gly (the glycosylated derivative of salicylic acid) 299 (93), ^2^H_5_-JA 214 (61), JA 209 (59), and JA-Ile/Leu 322 (130). The daughter masses were denoted in the brackets listed above. Each treatment had five replicates.

### 4.6. RNA Extraction and Gene Expression Quantification

The total RNA of the *Arabidopsis* leaves was extracted using TRIzol Reagent (Invitrogen, Carlsbad, MA, USA) as the described protocol. First-strand cDNA was synthesized using the Applied Biosystems reverse transcription kit (Applied Biosystem, Waltham, MA, USA, catalog number: 4368814). Gene expression was quantified using the real-time qPCR with the CFX96 real-time PCR detection system (Bio-Rad, Hercules, CA, USA). Data was analyzed using the 2^−∆*C*t^ method. *ACTIN* was used as the internal reference gene. The primers used in this study were listed in Table S1.

## Supplementary Materials

Supplementary materials can be found at https://www.mdpi.com/1422-0067/21/4/1223/s1. Figure S1: OX activated the plant disease resistance against the *Pst* DC3000 infection in tobacco (A) and tomato (B) plants. The tobacco or tomato plants were sprayed with OX (40 μg/mL) or mock (water with DMSO). The leaves were infiltrated with the *Pst* DC3000 (1 × 10^5^ CFU/mL, OD_600_ = 0.0002) using a 1 mL syringe two days after the spray treatment. Three days after the pathogen inoculation, the bacterial growth was quantified by the plate assay. Significant differences between the OX-treated plants and the mock were indicated by the asterisks determined from the Student’s *t*-test (*p* < 0.05). Results shown (means ± SD) are from one of the three independent repeats with the consistent results. Table S1: Primers used for qPCR in this study.

## References

[1] Savary S., Willocquet L., Pethybridge S.J., Esker P., McRoberts N., Nelson A. (2019). The global burden of pathogens and pests on major food crops. Nat. Ecol. Evol..

[2] Jones J.D., Dangl J.L. (2006). The plant immune system. Nature.

[3] Chisholm S.T., Coaker G., Day B., Staskawicz B.J. (2006). Host-Microbe Interactions: Shaping the Evolution of the Plant Immune Response. Cell.

[4] Katagiri F., Tsuda K. (2010). Understanding the Plant Immune System. Mol. Plant Microbe. Interact..

[5] Bittel P., Robatzek S. (2007). Microbe-associated molecular patterns (MAMPs) probe plant immunity. Curr. Opin. Plant Biol..

[6] He P., Shan L., Sheen J. (2007). Elicitation and suppression of microbe-associated molecular pattern-triggered immunity in plant-microbe interactions. Cell Microbiol..

[7] Newman M.A., Sundelin T., Nielsen J.T., Erbs G. (2013). MAMP (microbe-associated molecular pattern) triggered immunity in plants. Front. Plant Sci..

[8] Conrath U., Beckers G.J.M., Flors V., García-Agustín P., Jakab G., Mauch F., Newman M.-A., Pieterse C.M.J., Poinssot B., Pozo M.J. (2006). Priming: Getting Ready for Battle. Mol. Plant Microbe. Interact..

[9] Ryals J., Uknes S., Ward E. (1994). Systemic Acquired Resistance. Plant Physiol..

[10] Ryals J.A., Neuenschwander U.H., Willits M.G., Molina A., Steiner H.Y., Hunt M.D. (1996). Systemic Acquired Resistance. Plant Cell.

[11] Sticher L., Mauch-Mani B., Métraux J. (1997). SYSTEMIC ACQUIRED RESISTANCE. Annu. Rev. Phytopathol..

[12] Durrant W.E., Dong X. (2004). SYSTEMIC ACQUIRED RESISTANCE. Annu. Rev. Phytopathol..

[13] Cameron R.K., Paiva N.L., Lamb C.J., Dixon R.A. (1999). Accumulation of salicylic acid and *PR-1* gene transcripts in relation to the systemic acquired resistance (SAR) response induced by *Pseudomonas syringae* pv. *tomato* in *Arabidopsis*. Physiol. Mol. Plant Pathol..

[14] Kohler A., Schwindling S., Conrath U. (2002). Benzothiadiazole-Induced Priming for Potentiated Responses to Pathogen Infection, Wounding, and Infiltration of Water into Leaves Requires the *NPR1/NIM1* Gene in *Arabidopsis*. Plant Physiol..

[15] Barilli E., Sillero J.C., Rubiales D. (2010). Induction of Systemic Acquired Resistance in Pea against Rust (*Uromyces pisi*) by Exogenous Application of Biotic and Abiotic Inducers. J. Phytopathol..

[16] White R.F. (1979). Acetylsalicylic acid (aspirin) induces resistance to tobacco mosaic virus in tobacco. Virology.

[17] Vernooij B., Friedrich L., Morse A., Reist R., Kolditz-Jawhar R., Ward E., Uknes S., Kessmann H., Ryals J. (1994). Salicylic Acid Is Not the Translocated Signal Responsible for Inducing Systemic Acquired Resistance but Is Required in Signal Transduction. Plant Cell.

[18] Görlach J., Volrath S., Knauf-Beiter G., Hengy G., Beckhove U., Kogel K.H., Oostendorp M., Staub T., Ward E., Kessmann H. (1996). Benzothiadiazole, a novel class of inducers of systemic acquired resistance, activates gene expression and disease resistance in wheat. Plant Cell.

[19] Lawton K.A., Friedrich L., Hunt M., Weymann K., Delaney T., Kessmann H., Staub T., Ryals J. (1996). Benzothiadiazole induces disease resistance in *Arabidopsis* by activation of the systemic acquired resistance signal transduction pathway. Plant J..

[20] Benhamou N., Bélanger R.R. (1998). Benzothiadiazole-Mediated Induced Resistance to *Fusarium oxysporum* f. sp. *radicis-lycopersici* in Tomato. Plant Physiol..

[21] Iriti M., Rossoni M., Borgo M., Faoro F. (2004). Benzothiadiazole Enhances Resveratrol and Anthocyanin Biosynthesis in Grapevine, Meanwhile Improving Resistance to *Botrytis cinerea*. J. Agric. Food Chem..

[22] Zimmerli L., Métraux J.-P., Mauch-Mani B. (2001). *β*-Aminobutyric Acid-Induced Protection of *Arabidopsis* against the Necrotrophic Fungus *Botrytis cinerea*. Plant Physiol..

[23] Zimmerli L., Jakab G., Métraux J.-P., Mauch-Mani B. (2000). Potentiation of pathogen-specific defense mechanisms in *Arabidopsis* by *β*-aminobutyric acid. Proc. Natl. Acad. Sci. USA.

[24] Hamiduzzaman M.M., Jakab G., Barnavon L., Neuhaus J.M., Mauch-Mani B. (2005). beta-Aminobutyric acid-induced resistance against downy mildew in grapevine acts through the potentiation of callose formation and jasmonic acid signaling. Mol. Plant Microbe Interact..

[25] Li T., Fan P., Yun Z., Jiang G., Zhang Z., Jiang Y. (2019). *β*-Aminobutyric Acid Priming Acquisition and Defense Response of Mango Fruit to *Colletotrichum gloeosporioides* Infection Based on Quantitative Proteomics. Cells.

[26] Cordier C., Pozo M.J., Barea J.M., Gianinazzi S., Gianinazzi-Pearson V. (1998). Cell Defense Responses Associated with Localized and Systemic Resistance to *Phytophthora parasitica* Induced in Tomato by an Arbuscular Mycorrhizal Fungus. Mol. Plant Microbe Interact..

[27] Pozo M.J., Cordier C., Dumas-Gaudot E., Gianinazzi S., Barea J.M., Azcón-Aguilar C. (2002). Localized versus systemic effect of arbuscular mycorrhizal fungi on defence responses to *Phytophthora* infection in tomato plants. J. Exp. Bot..

[28] Xin X.-F., Kvitko B., He S.Y. (2018). *Pseudomonas syringae*: What it takes to be a pathogen. Nat. Rev. Microbiol..

[29] Hirano S.S., Upper C.D. (2000). Bacteria in the Leaf Ecosystem with Emphasis on *Pseudomonas syringae*—A Pathogen, Ice Nucleus, and Epiphyte. Microbiol. Mol. Biol. Rev..

[30] Cuppels D.A. (1986). Generation and characterization of Tn5 insertion mutations in *Pseudomonas syringae* pv. tomato. Appl. Environ. Microbiol..

[31] Xin X.F., He S.Y. (2013). *Pseudomonas syringae* pv. *tomato* DC3000: A model pathogen for probing disease susceptibility and hormone signaling in plants. Annu. Rev. Phytopathol..

[32] Pasteris R., Hanagan M., Shapiro R.J.I. (2008). Geneva: World Intellectual Property Organization, Fungicidal Azocyclic Amides.

[33] Pasteris R.J., Hanagan M.A., Bisaha J.J., Finkelstein B.L., Hoffman L.E., Gregory V., Andreassi J.L., Sweigard J.A., Klyashchitsky B.A., Henry Y.T. (2016). Discovery of oxathiapiprolin, a new oomycete fungicide that targets an oxysterol binding protein. Bioorgan. Med. Chem..

[34] Ji P., Csinos A.S., Hickman L.L., Hargett U. (2014). Efficacy and application methods of oxathiapiprolin for management of black shank on tobacco. Plant Dis..

[35] Cohen Y. (2015). The novel oomycide oxathiapiprolin inhibits all stages in the asexual life cycle of *Pseudoperonospora cubensis*-causal agent of cucurbit downy mildew. PLoS ONE.

[36] Ji P., Csinos A.S. (2015). Effect of oxathiapiprolin on asexual life stages of *Phytophthora capsici* and disease development on vegetables. Ann. Appl. Biol..

[37] Miao J., Dong X., Lin D., Wang Q., Liu P., Chen F., Du Y., Liu X. (2016). Activity of the novel fungicide oxathiapiprolin against plant-pathogenic oomycetes. Pest. Manag. Sci..

[38] Torres M.A., Dangl J.L., Jones J.D. (2002). *Arabidopsis* gp91phox homologues *AtrbohD* and *AtrbohF* are required for accumulation of reactive oxygen intermediates in the plant defense response. Proc. Natl. Acad. Sci. USA.

[39] Luna E., Pastor V., Robert J., Flors V., Mauch-Mani B., Ton J. (2011). Callose deposition: A multifaceted plant defense response. Mol. Plant.

[40] Jin L., Mackey D.M. (2017). Measuring Callose Deposition, an Indicator of Cell Wall Reinforcement, During Bacterial Infection in *Arabidopsis*. Methods Mol. Biol..

[41] Penninckx I.A.M.A., Thomma B.P.H.J., Buchala A., Métraux J.-P., Broekaert W.F. (1998). Concomitant Activation of Jasmonate and Ethylene Response Pathways Is Required for Induction of a Plant Defensin Gene in *Arabidopsis*. Plant Cell.

[42] Quirino B.F., Bent A.F. (2003). Deciphering host resistance and pathogen virulence: The *Arabidopsis/Pseudomonas* interaction as a model. Mol. Plant Pathol..

[43] Qi J., Wang J., Gong Z., Zhou J.-M. (2017). Apoplastic ROS signaling in plant immunity. Curr. Opin. Plant Biol..

[44] Hirt H. (2016). Aquaporins Link ROS Signaling to Plant Immunity. Plant Physiol..

[45] Kadota Y., Shirasu K., Zipfel C. (2015). Regulation of the NADPH Oxidase RBOHD During Plant Immunity. Plant Cell Physiol..

[46] Malinovsky F.G., Fangel J.U., Willats W.G. (2014). The role of the cell wall in plant immunity. Front. Plant Sci..

[47] Hückelhoven R. (2007). Cell Wall–Associated Mechanisms of Disease Resistance and Susceptibility. Annu. Rev. Phytopathol..

[48] Verma V., Ravindran P., Kumar P.P. (2016). Plant hormone-mediated regulation of stress responses. BMC Plant Biol..

[49] Bari R., Jones J.D.G. (2009). Role of plant hormones in plant defence responses. Plant Mol. Biol..

[50] Spoel S.H., Dong X. (2008). microbe, Making sense of hormone crosstalk during plant immune responses. Cell Host Microbe.

[51] Kunkel B.N., Brooks D.M. (2002). Cross talk between signaling pathways in pathogen defense. Curr. Opin. Plant Biol..

[52] Beckers G.J.M., Spoel S.H. (2006). Fine-Tuning Plant Defence Signalling: Salicylate versus Jasmonate. Plant Biol. (Stuttg).

[53] Jia X., Zeng H., Wang W., Zhang F., Yin H. (2018). Chitosan Oligosaccharide Induces Resistance to *Pseudomonas syringae* pv. *tomato* DC3000 in *Arabidopsis thaliana* by Activating Both Salicylic Acid- and Jasmonic Acid-Mediated Pathways. Mol. Plant Microbe Interact..

[54] Melotto M., Underwood W., Koczan J., Nomura K., He S.Y. (2006). Plant Stomata Function in Innate Immunity against Bacterial Invasion. Cell.

[55] Iriti M., Rossoni M., Borgo M., Ferrara L., Faoro F. (2005). Induction of Resistance to Gray Mold with Benzothiadiazole Modifies Amino Acid Profile and Increases Proanthocyanidins in Grape: Primary versus Secondary Metabolism. J. Agric. Food Chem..

[56] Cohen Y.R. (2002). *β*-Aminobutyric Acid-Induced Resistance Against Plant Pathogens. Plant Dis..

[57] Zipfel C., Robatzek S., Navarro L., Oakeley E.J., Jones J.D.G., Felix G., Boller T. (2004). Bacterial disease resistance in *Arabidopsis* through flagellin perception. Nature.

[58] Zhang J., Li W., Xiang T., Liu Z., Laluk K., Ding X., Zou Y., Gao M., Zhang X., Chen S. (2010). Receptor-like cytoplasmic kinases integrate signaling from multiple plant immune receptors and are targeted by a *Pseudomonas syringae* effector. Cell Host Microbe.

[59] Wang M., Rui L., Yan H., Shi H., Zhao W., Lin J.E., Zhang K., Blakeslee J.J., Mackey D., Tang D. (2018). The major leaf ferredoxin Fd2 regulates plant innate immunity in *Arabidopsis*. Mol. Plant. Pathol..

[60] Forcat S., Bennett M.H., Mansfield J.W., Grant M.R. (2008). A rapid and robust method for simultaneously measuring changes in the phytohormones ABA, JA and SA in plants following biotic and abiotic stress. Plant Methods.

