# Supplementary material for: Oxathiapiprolin, a Novel Chemical Inducer Activates the Plant Disease Resistance
Source: Int J Mol Sci. 2020 Feb 12;21(4):1223. doi: 10.3390/ijms21041223 (PMC7072870; doi:10.3390/ijms21041223)
Supplement: Supplementary file 1 [file ijms-21-01223-s001.pdf]

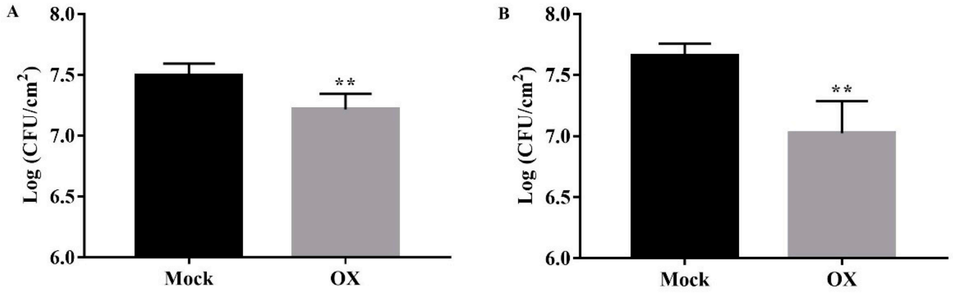

Figure S1: OX activated the plant disease resistance against the *Pst* DC3000 infection in tobacco (A) and tomato (B) plants. The tobacco or tomato plants were sprayed with OX (40 µg/ml) or mock (water with DMSO). The leaves were infiltrated with the *Pst* DC3000 (1×10^5 CFU/ml, $OD_{600} = 0.0002$) using a 1 ml syringe at two days post the spray treatment. Three days after the pathogen inoculation, the bacterial growth was quantified by the plate assay. Significant differences between the OX-treated plants and the mock were indicated by the asterisks determined from the Student's t test ($P < 0.05$). Results shown (means ± SD) are from one of the three independent repeats with the consistent results.

Table S1 Primers used for qPCR in this study

Gene	Forward primer	Reverse primer
PR1	TTCTTCCCTCGAAAGCTCAA	AAGGCCCACCAGAGTGTATG
PR2	CATCCTCGACGTTCCCAGTT	TGTCGGCCTCCGTTTGA
PR5	AACGGCGGCGGAGTTC	GCCGCCATCGCCTACTAGA
PDF 1.2	GTTTGCGGAAACAGTAATGC	CACACGATTTAGCACCAAAGA
RBOHD	AGCTTCACAATTATTGC ACGAG	TCTCCAGTTAGGTTTA GCGAAG
ABI4	CCGCTTCTTCTCCTTCCAC	GAGGGAGGAGAGGTCTTAGGG
ABA2	ACTCGCTTTGGCTCATTTGCC	ACCGTCAGTTCCACCCCTTT
ACTIN	AGTGGTCGTACAACCGGTATTGT	GAGGAAGAGCATACCCCTCGTA
